# Adenovirus-Mediated Efficient Gene Transfer into Cultured Three-Dimensional Organoids

**DOI:** 10.1371/journal.pone.0093608

**Published:** 2014-04-02

**Authors:** Ning Wang, Hongyu Zhang, Bing-Qiang Zhang, Wei Liu, Zhonglin Zhang, Min Qiao, Hongmei Zhang, Fang Deng, Ningning Wu, Xian Chen, Sheng Wen, Junhui Zhang, Zhan Liao, Qian Zhang, Zhengjian Yan, Liangjun Yin, Jixing Ye, Youlin Deng, Hue H. Luu, Rex C. Haydon, Houjie Liang, Tong-Chuan He

**Affiliations:** 1 Department of Oncology and Southwest Cancer Center, the Affiliated Southwest Hospital, Third Military Medical University, Chongqing, China; 2 Molecular Oncology Laboratory, Department of Surgery, The University of Chicago Medical Center, Chicago, Illinois, United States of America; 3 Ministry of Education Key Laboratory of Clinical Diagnostic Medicine, and the Affiliated Hospitals of Chongqing Medical University, Chongqing, China; 4 Department of Surgery, Affiliated Zhongnan Hospital of Wuhan University, Wuhan, China; 5 Department of Orthopaedic Surgery, Xiang-Ya Hospital of Central South University, Changsha, China; 6 School of Bioengineering, Chongqing University, Chongqing, China; Swedish Medical Center, United States of America

## Abstract

Three-dimensional organoids have been recently established from various tissue-specific progenitors (such as intestinal stem cells), induced pluripotent stem cells, or embryonic stem cells. These cultured self-sustaining stem cell–based organoids may become valuable systems to study the roles of tissue-specific stem cells in tissue genesis and disease development. It is thus conceivable that effective genetic manipulations in such organoids may allow us to reconstruct disease processes and/or develop novel therapeutics. Recombinant adenoviruses are one of the most commonly used viral vectors for *in vitro* and *in vivo* gene deliveries. In this study, we investigate if adenoviruses can be used to effectively deliver transgenes into the cultured “mini-gut” organoids derived from intestinal stem cells. Using adenoviral vectors that express fluorescent proteins, we demonstrate that adenoviruses can effectively deliver transgenes into the cultured 3-D “mini-gut” organoids. The transgene expression can last at least 10 days in the cultured organoids. As a proof-of-principle experiment, we demonstrate that adenovirus-mediated noggin expression effectively support the survival and self-renewal of mini-gut organoids, while adenovirus-mediated expression of BMP4 inhibits the self-sustainability and proliferation of the organoids. Thus, our results strongly suggest that adenovirus vectors can be explored as effective gene delivery vehicles to introduce genetic manipulations in 3-D organoids.

## Introduction

The epithelial lining of the small intestine is a multifunctional tissue and renewed at an extraordinary rate (with a turnover time less than 5 days) throughout life in the vertebrate body [1], [2]. This process is driven by small populations of adult stem cells (also known as intestine stem cells) that reside within specialized niches. Intestinal stem cells reside near the bottom of the intestinal crypt. The stem cells give rise to rapidly dividing, transit-amplifying daughter cells that occupy the remainder of the crypts and flow onto the flanks of the villi, where they differentiate into absorptive enterocytes, multiple secretory cells (Paneth cells, goblet cells, enteroendocrine cells, and tuft cells), and the M cells of Peyer's patches [2]. These stem cells can undergo indefinite self-renewal and generate new functional epithelia, rendering themselves valuable sources for studying intestine tissue homeostasis, tissue genesis, and development of disease models.

Recent studies have shown that single intestinal Lgr5^+^ stem cells can grow into structures that faithfully recapitulate the self-renewing 3-dimensional (3-D) intestinal epithelium structures, called “mini-gut” organoids [3]–[9]. Organoids recapitulate the intestine stem cell differentiation hierarchy and allow the in vitro study of cell fate determination. Organoid technology is well suited to the study of biological phenomena that require a closed epithelial structure with a physiological topology [2], [6].

In fact, it has been reported that 3-dimensional organoids derived from tissue-specific progenitors, induced pluripotent stem cells, or embryonic stem cells have been reported for small intestine, colon, stomach, pancreas [3]–[11], liver [12]–[14], kidney [15], and cerebral organoids [16]. These self-organizing organoid systems should become valuable tools to study the roles of tissue-specific stem cells in tissue genesis and disease development. It is conceivable that effective gene manipulations, including overexpression, knockdown, and gene editing with TALEN and CRISPR systems [17]–[21], in such organoids may allow us to reconstruct disease processes and/or develop novel therapeutics. Thus, there is a great need to develop quick, efficient, and simplistic methods to facilitate genetic manipulations in these cultured organoids.

Recombinant adenoviruses are one of the most commonly used approaches for efficient in vitro and in vivo gene deliveries [22]–[26]. Some noted advantages for adenovirus-mediated gene delivery include the ease of obtaining high titers, viral particle stability, large packaging capacity of foreign DNA, high level of transgene expression, and the ability to transduce a wide range of tissues and cells including nondividing cells [22], [26]. The non-integration feature in host chromosomes is also considered advantageous as it poses no disturbances in genes or cellular processes at the genome within the body.

Here, we investigate if adenovirus can be used to effectively deliver transgenes into cultured mini-gut organoids. Using adenoviral vectors that express fluorescent proteins, we demonstrate that recombinant adenoviruses can effectively deliver transgenes into the 3-D “mini-gut” organoid culture. The transgene expression can last at least 10 days in the cultured organoids. It has been well established that noggin is essential to maintain the self-sustainability of gut stem cells, which is antagonized by BMP signaling [27]–[29]. As a proof-of-principle experiment, we demonstrate that adenovirus-mediated noggin expression effectively support the survival and self-renewal of mini-gut organoids, while adenovirus-mediated expression of BMP4 inhibits the self-sustainability and proliferation of the organoids. Thus, our results strongly suggest that adenovirus vectors can be explored as effective gene delivery vehicles to introduce genetic manipulations in 3-D organoids derived from tissue-specific stem cells, induced pluripotent stem cells, and/or embryonic stem cells.

## Materials and Methods

### Cell culture and chemicals

HEK-293 cells were purchased from ATCC (Manassas, VA) and maintained in the completed DMEM medium as described [30]–[33]. Organoid culture medium consisted of the “mini-gut medium” and ENR growth factors as described [3]–[9]. The “mini-gut medium” was based on the Advanced DMEM/F12 medium (Invitrogen), supplemented with 1x N2 supplement (Invitrogen), 1x B27 supplement (Invitrogen), penicillin/streptomycin (P/S, each at 100 unit/ml), 2 mM L-glutamine, 10 mM HEPES (pH 7.4). ENR growth factor medium contained 50 ng/ml EGF (Sigma-Aldrich), 100 ng/ml noggin (PeproTech Co.), Rspo2 conditioned medium (10% v/v). Rho kinase (ROCK) inhibitor Y27632 (10 μM, Selleck Chemicals, Houston, TX) was also added to ENR medium. ENR growth factors were mixed freshly before adding to “mini-gut medium”. Growth factor reduced Matrigel was purchased from BD Biosciences. Unless indicated otherwise, all chemicals were purchased from Sigma-Aldrich (St. Louis, MO) or Fisher Scientific (Pittsburgh, PA).

### Construction and generation of recombinant adenoviruses expressing noggin, BMP4, GFP, and/or RFP

Recombinant adenoviruses were generated using AdEasy technology as described [34]–[38]. The coding region of human BMP4, mouse noggin, green fluorescence protein (GFP), or monomeric red fluorescence protein (RFP) was PCR amplified and cloned into an adenoviral shuttle vector, which was subsequently used to generate recombinant adenoviruses in HEK-293 cells. The resulting adenoviruses were designated as Ad-BMP4, Ad-Noggin, Ad-GFP and Ad-RFP. Ad-BMP4 also expresses GFP [34], [36], [37] while Ad-Noggin also expresses RFP [39].

### Preparation of R-spondin 2 (Rspo2) conditioned medium

Stable mouse R-spondin2 expression line 293-Rspo2 was kindly provided by Dr. Jeffrey A. Whitsett of University of Cincinnati Children's Hospital. Cells were cultured in complete DMEM (150 cm^2^ flask) and allowed to reach 90% confluence. DMEM was removed, washed with PBS twice, replaced with 30 ml OPTI-MEM (Invitrogen). Rspo2 conditioned medium was collected at day 4, followed by another collection at day 8. Both batches of conditioned medium were mixed, aliquoted, and kept at −80°C. The biological activity of Rspo2 conditioned medium was monitored by assessing its ability to activate Wnt/β-catenin pathway using the Top-flash firefly luciferase reporter assay[31], [32], [40]–[43].

### Isolation of mouse intestinal crypts and establishment of 3-dimensional “mini-gut” organoids

All animal experiments reported in this study were carried out in strict accordance with the recommendations in the Guide for the Care and Use of Laboratory Animals of the National Institutes of Health. The protocol was approved by the Institutional Animal Care and Use Committee (IACUC) of The University of Chicago (protocol Number #71108). Briefly, young CD1 mice (2 to 3-week old, male) were obtained from The University of Chicago Transgenic Core Facility. After the animals were euthanized, the dissected small intestines (mostly dissected jejunum and ileum) were opened longitudinally, washed with cold PBS (with P/S), and cut into 0.5–1.0 cm pieces. The tissues were rocked in PBS with 2 mM EDTA for 30 minutes at 4°C, and then switched to PBS with 54.9 mM D-sorbitol and 43.4 mM sucrose, followed by vortexing for 1–2 minutes and filtering through 100 μm sterile cell strainers. The crypts were collected by centrifugation at 150 g×4 min 4°C. Approximately 500 crypts were mixed with 100 μl per well of Matrigel, plated in 24-well plates, and incubated for 30 minutes in a 37°C 5% CO_2_ incubator to allow the Matrigel to polymerize. 500 μl of organoid culture medium were then added to each well. The medium was changed every three days. For passage, organoids were removed from Matrigel and mechanically dissociated into single-crypt domains by passing through a 1 cc syringe/needle (27G, BD Biosciences), and then transferred to fresh Matrigel. Passage was performed every 7–10 days with a 1∶4 split ratio.

### Adenovirus infection of cultured organoids

We developed two infection protocols. The first approach was called “mix and seed”. When organoids were ready for splitting, aliquots of the pre-titrated adenovirus were added to Matrigel mixtures, gently mixed well and then seeded into multi-well culture plates. The second approach was called “incubate and seed”. In this approach, adenovirus aliquots were directly mixed with resuspended organoids in 100 μl organoid culture medium with occasional gentle agitation in a 37°C 5% CO_2_ incubator for 30 min. The infected organoids were briefly spun down, mixed with 50–100 μl ice-chilled Matrigel, and then seeded onto 24-well culture plates. In both approaches, the organoid culture medium was changed every three days.

For quantitative analysis of actively growing organoids, at the indicated time point the cultured organoids were examined under high power fields. The numbers of actively growing organoids, which exhibited at least two budding structures, over total organoids in each group, were counted. At least 100 random organoids were examined from each group which was initially seeded in triplicate. The percentage of growing organoids for each treatment was expressed as mean ± SD%.

### Statistical analysis

All quantitative experiments were performed in triplicate and/or repeated three times. Data were expressed as mean±SD. Statistical significances between groups were determined by one-way analysis of variance and the Student's *t* test. A value of *p*<0.05 was considered statistically significant.

## Results and Discussion

### Establishment of mouse intestinal crypt stem cell-containing, self-sustaining “mini-gut” organoids

**Figure 1:**
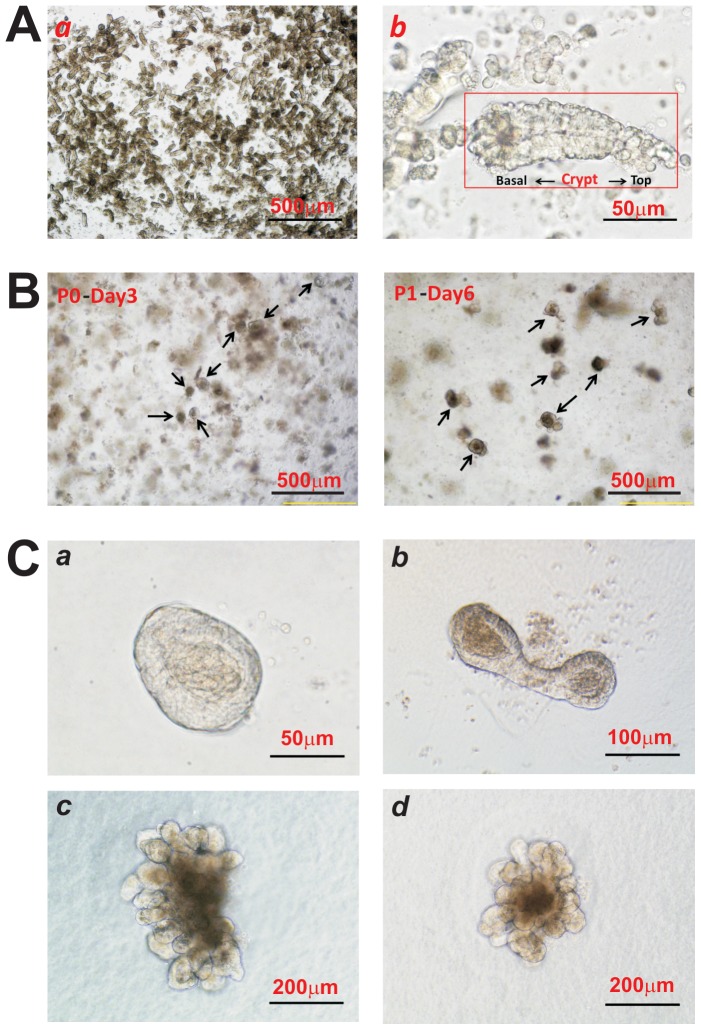
Establishment of mouse intestinal crypt stem cell-containing, self-sustaining organoids. (**A**) Isolation of intestinal crypt cells. The intestinal crypt from the jejunum and ileum of young CD1 mice were harvested as described in Methods (***a***). Some intact crypt structures were also retained during the isolation process (***b***). (**B**) The isolated crypt cells were seeded in Matrigel and maintained in the organoid culture medium as described in Methods. The proliferation of crypt cells and organoid formation were apparent at day 3 while budding organoids were readily observed at day 6 after seeding. (**C**) Representative images of various forms of crypt organoids from the passed crypt culture are shown.

**Figure 2:**
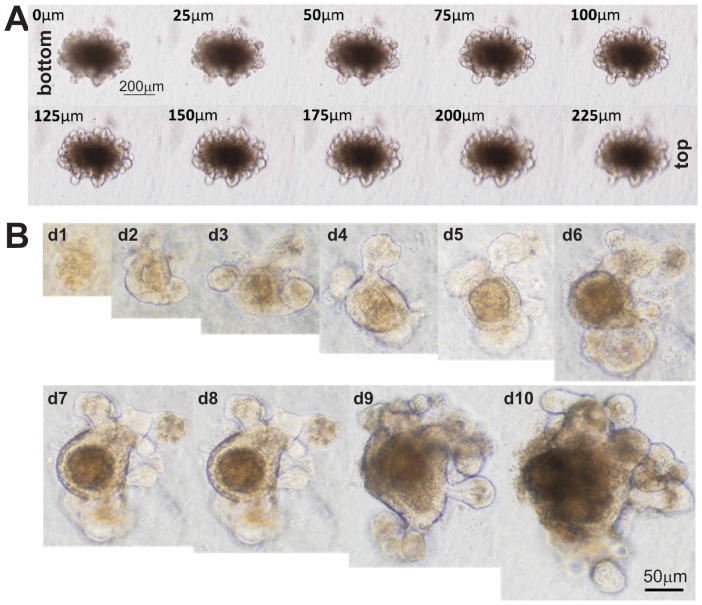
Three-dimensional morphology of the cultured “mini-gut” organoids. (**A**) Z-stack serial images of a multi-budding organoid. The bottom-to-top distance was 225 μm with 25 μm distance interval between images. (**B**) Monitoring the growth of a single organoid over a period of 10 days in 3-D Matrigel culture. Representative images are shown.

Following the reported procedures [3]–[9], we harvested intestinal crypts from jejunum and ileum of young CD1 mice (**Figure 1A**). The isolated intestinal crypt cells were seeded in Matrigel and maintained in organoid culture medium [9]. The proliferation of crypt cells and organoid formation were apparent at day 3 while budding organoids were readily observed at day 6 after seeding (**Figure 1B**). The cultured organoids can be passaged for several months as self-sustaining organoids (**Figure 1C**) and form multi-budding “mini-gut” 3-D organoid structures (**Figure 2**).

### Adenovirus-mediated efficient transgene delivery into cultured “mini-gut” organoids

We next tested if the cultured organoids could be efficiently transduced by recombinant adenoviruses. To carry out the proof-of-concept experiments, we used the adenoviruses Ad-GFP and Ad-RFP, which express GFP and RFP, respectively [34], [35], [37], [38], which allowed us to effectively monitor the efficiency and duration of adenovirus-mediated gene delivery. In our initial attempts, we found that a direct addition of adenoviruses (at a broad range of dosages) to the medium of the Matrigel organoid culture failed to yield any significant gene transfer (data not shown). Thus, the cultured organoids were infected using two different approaches in order to maximize the infection efficiency while minimizing any detrimental effects on the survival of the infected organoids.

**Figure 3:**
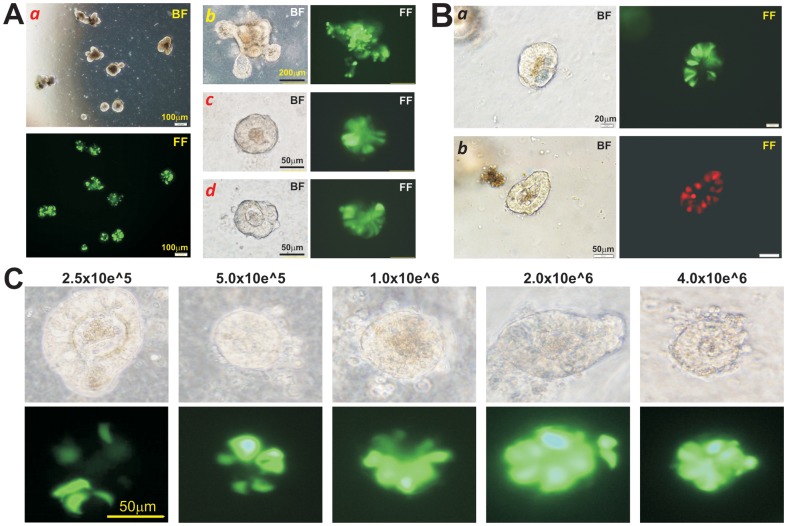
Adenovirus-mediated efficient transgene delivery into cultured “mini-gut” organoids. Two general approaches were used to infect the cultured organoids. (**A**) “Mix and seed” approach. When organoids were ready for splitting, an aliquot of the pre-titrated adenovirus, e.g., Ad-GFP in this case at 5×10^5^ pfu per 100 μl organoid culture, was added to the Matrigel, gently mixed well and the seeded into multiple well culture plates. Adenovirus-mediated GFP expression was observed at 24 h, at low magnification (***a***) or at higher magnification (***b–d***). (**B**) “Incubate and seed” approach. In this approach, Ad-GFP or Ad-RFP aliquots were directly mixed with resuspended organoids in 100 μl organoid culture medium with occasional gentle agitation in a 37°C CO_2_ incubator for 30 min. The infected organoids were briefly spun down, mixed with 50–100 μl ice-chilled Matrigel, and then seeded onto 24-well culture plates. Fluorescence signal was examined and recorded after 24 h (***a*** & ***b***). (**C**) Dose-dependent gene transfer into the organoids. The resuspended organoids were incubated with the indicated titers of Ad-GFP in a 37°C CO_2_ incubator for 30 min, mixed with 100 μl cold Matrigel, and seeded into 24-well culture plates. GFP signal was detected at 48 h after seeding. All fluorescence images were obtained under the same exposure condition. Representative results are shown. BF, bright field; FF, fluorescence field.

In the “mix and seed” approach, we added and mixed the pre-titrated Ad-GFP (5×10^5^ pfu per 100 μl organoid culture, in less than 5 μl) with Matrigel mix containing organoids, and seeded to multi-well culture plates. GFP expression was readily detected at 24 h after seeding (**Figure 3A**). We also used the alternative “incubate and seed” approach, in which adenovirus (Ad-GFP or Ad-RFP) aliquots were mixed with resuspended organoids (in 100 μl organoid culture medium) and incubated in the 37°C CO_2_ incubator for 30 min. The infected organoids were then briefly spun down, mixed with 100 μl ice-chilled Matrigel, and then seeded in multi-well culture plates. Adenovirus-mediated expression of GFP or RFP was readily detected at 24 h after seeding (**Figure 3B**). In fact, the transduced organoids were shown high levels of fluorescent protein expression (**Video S1**). Both infection approaches were shown to be effective and reproducible in terms of infection efficiency. We found that the “mix and seed” method is more simplistic and better for organoid survival while the “incubate and seed” approach achieves more uniform infection of the organoids.

We also tested the dosage-dependence and cytotoxicity of adenovirus infection. Using the “incubate and seed” method, we tested a broad range of adenovirus titers (2.5×10^5^ to 4.0×10^6^ pfu per 100 μl organoid culture) for their transduction efficiency and toxicity. At 48 h after seeding of the infected organoids, all infected organoids exhibited significant levels of GFP signal. Among the tested titers, 5.0×10^5^ pfu yielded the best result (with high GFP expression but no significant cytopathic effects) while significant toxicities were observed in the organoid culture incubated with higher than 1.0×10^6^ pfu Ad-GFP (**Figure 3C**). Given the fact that the cell numbers in each organoid culture are usually estimated, it is relatively difficult to calculate the exact multiplicities of infection (MOIs) in these experiments. Based on our experience, we estimate that MOIs of 50–100 may be required for effective adenovirus transduction of the organoids. Nonetheless, we found that most cultured organoids could tolerate a rather broad range of adenovirus titers, and thus adenovirus-mediated gene transduction can still be practiced reproducibly.

**Figure 4:**
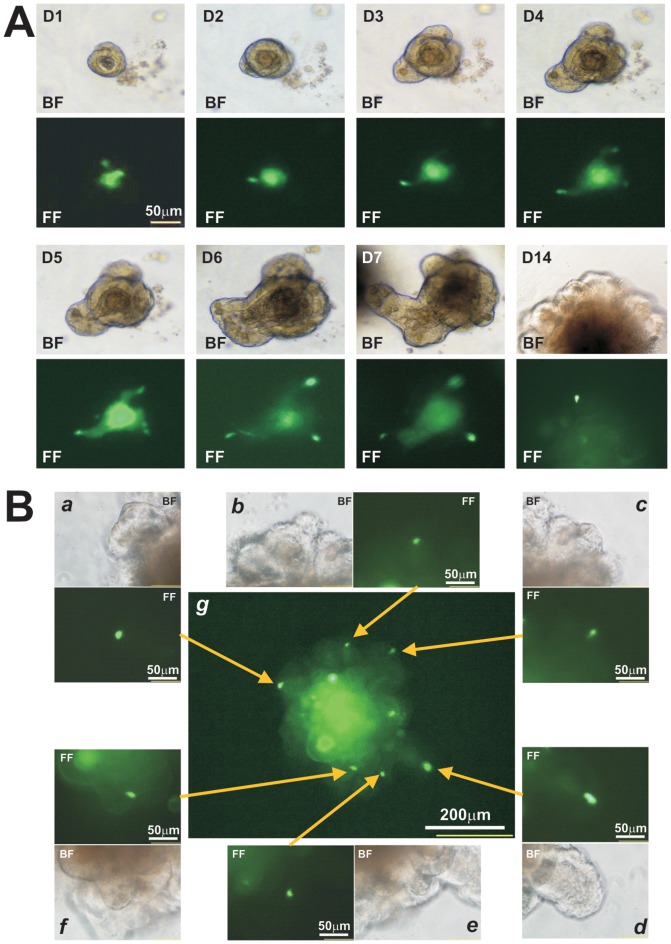
Duration of adenovirus-transduced gene expression in cultured organoids. (**A**) Time course of adenovirus-mediated GFP expression. 5×10^5^ pfu Ad-GFP viral particles were directly added to the ice-chilled organoids Matrigel mix. Single infected organoids were followed for GFP expression for up to 14 days after infection. Time-course GFP expression of a representative organoid is shown. (**B**) GFP signal-retaining cells in the transduced organoids. The organoids were infected with Ad-GFP for 7 days (***g***). Some intense GFP-containing cells were spotted at budded organoids (***a*** to ***f***). Representative results are shown. BF, bright field; FF, fluorescence field.

We next tested how long the adenovirus-mediated gene transfer would last in cultured organoids. Using the “mix and seed” approach, we infected the organoids with Ad-GFP (at 5×10^5^ pfu per 100 μl organoid culture) and seeded in multi-well plates. Individually infected organoids were followed for GFP expression for up to 14 days after seeding. GFP signal was readily detectable up to 7 days in most of the infected organoids (**Figure 4A**). We noticed that GFP expression reached peaks at 5–7 days and significantly reduced in most organoids at 14 days after seeding while a couple of intense GFP positive spots (or cells) remained in some organoids (**Figure 4B**). We did not have evidence demonstrating if they represented quiescent progenitor-like cells. Nonetheless, our results indicate that robust exogenous gene expression can last at least 7 days in adenovirus-infected organoids.

### Adenovirus-mediated noggin expression sustains the survival of mini-gut organoids, which is inhibited by exogenous BMP4 expression

**Figure 5:**
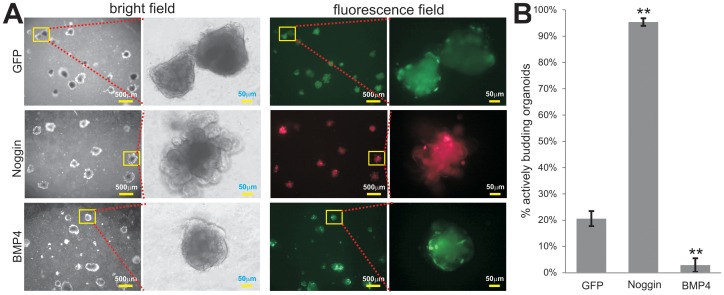
Adenovirus-mediated noggin expression sustains the survival of mini-gut organoids, which is inhibited by exogenous BMP4 expression. (A) 5×10^5^ pfu viral particles of Ad-Noggin (also expressing RFP), Ad-BMP4 (also expressing GFP), Ad-GFP or Ad-RFP were directly added to the ice-chilled organoids Matrigel mix. The infected organoids were cultured in the “mini-gut” medium containing all necessary factors but noggin (e.g., EGF, R-Spondin1, and the ROCK inhibitor), and examined under bright field and fluorescence microscopy at 6 days after infection. Representative results are shown. (B) Quantitative analysis of actively growing organoids upon noggin or BMP4 expression. At day 6 after infection the organoids infected with Ad-GFP, Ad-Noggin, or Ad-GFP were examined under high power fields to calculate the percentages of actively growing organoids (which exhibited at least two budding structures) over total organoids in each group. At least 100 organoids were counted in each treatment group. “**”, *p<0.001*.

We further conducted a proof-of-principle experiment to test if adenovirus-mediated noggin expression could provide the self-sustainability of the organoids, which should be inhibited by BMPs [29]. We infected the organoids with the same titer of Ad-Noggin (also expressing RFP), Ad-BMP4 (also expressing GFP), Ad-GFP or Ad-RFP. The infection efficiency was assessed at 48 h after seeding (**Figure S1**). The infected organoids were maintained in the “mini-gut” medium containing all necessary factors except Noggin (e.g., EGF, R-spondin2, and ROCK inhibitor). At 6 days after seeding, the organoids were examined under bright field and fluorescence microscopy. The presence of the infected organoids was readily detected in all three groups (**Figure 5A**). However, the Ad-Noggin infected organoids exhibited significantly more organoid budding than the Ad-GFP infected control group, while the Ad-BMP4 transduced organoids rarely had budding organoids (**Figure 5A**). When budding organoids were quantitatively tabulated under higher magnifications, 20.6±2.85% budding organoids were observed in the Ad-GFP group whereas 95.4±1.46% budding organoids were obtained in the Ad-Noggin group (**Figure 5B**). However, only less than 2.9±2.61% budding organoids were detected in the Ad-BMP4 treated group. These results demonstrate that adenovirus-mediated noggin expression can effectively compensate the noggin deficiency in the organoid culture medium, and that adenovirus-mediated BMP4 expression significantly inhibits the proliferation of mini-gut organoids [29]. Thus, these functional assays strongly suggest that adenovirus-mediated gene transfer may be used to investigate the roles of biological factors or signaling molecules in regulating physiological and/or pathological processes in tissue-specific stem cells.

### Adenovirus-mediated gene transfer into the 3-D organoids provides an efficient approach to genetic manipulations of stem cell-based self-organizing tissue-like structures

The use of adenovirus vectors in 3-D organoid culture offers several unparalleled advantages, compared with other gene delivery approaches including retroviral or lentiviral vectors [5]. First, adenovirus vectors can achieve high titers and achieve efficient transgene expression. Second, organoids can be easily re-infected with the same adenovirus in the organoid culture setting. Third, organoids can be co-infected with adenoviruses expressing different factors. Fourth, if necessary a temporal delivery of multiple factors can be achieved by adenovirus infections at different time points. Fifth, for the organoids that are isolated from conditional knockout/transgenic animals, adenoviral vectors can be used to effectively deliver Cre or FLP recombinase into the intestinal stem cells. Lastly, the transient but efficient nature of adenovirus-mediated gene transfer can be used to directly deliver cutting-edge genome editing systems, such as TALEN and CRISPR [17]–[21], into tissue-specific progenitor cells or stem cells. These features would allow us to directly manipulate normal progenitors/stem cells so that organoids may not need to be derived from knockout and/or transgenic animals. The organoids may become valuable systems to study the roles of tissue-specific stem cells in tissue genesis and disease development. Ultimately, effective gene manipulations in such organoids should allow us to reconstruct disease processes and/or develop novel therapeutics.

In summary, we demonstrate that recombinant adenoviruses can effectively deliver transgenes into the 3-D “mini-gut” organoid culture. The transgene expression can last at least 10 days in the cultured organoids. As a proof-of-principle experiment, we demonstrate that adenovirus-mediated noggin expression effectively support the survival and self-renewal of mini-gut organoids, while adenovirus-mediated expression of BMP4 inhibits the self-sustainability and proliferation of the organoids. Thus, our results strongly suggest that adenovirus vectors can be explored as effective gene delivery vehicles to introduce genetic manipulations in 3-D organioids derived from tissue-specific stem cells, induced pluripotent stem cells, and/or embryonic stem cells.

## Supporting Information

Figure S1 **Efficient transduction of mini-gut organoids by adenoviral vector expressing noggin, BMP4, GFP, and/or RFP.** 5×10^5^ pfu viral particles of Ad-Noggin (also expressing RFP), Ad-BMP4 (also expressing GFP), and Ad-RFP were added to the ice-chilled organoids Matrigel mix. The infected organoids were followed for fluorescence signal at 48 h after infection. Representative results are shown.
(TIF)

Video S1 **The organoids were infected with Ad-GFP for 24 h and then subjected to a 24 h-time-lapse fluorescence imaging.** Up to 10 organoids were imaged simultaneously. Images were taken every 301min and synthesized into movie files. A representative imaged organoid is shown.
(MP4)
