# Correction: Adenovirus-Mediated Efficient Gene Transfer into Cultured Three-Dimensional Organoids

**DOI:** 10.1371/journal.pone.0259544

**Published:** 2021-10-28

**Authors:** Ning Wang, Hongyu Zhang, Bing-Qiang Zhang, Wei Liu, Zhonglin Zhang, Min Qiao, Hongmei Zhang, Fang Deng, Ningning Wu, Xian Chen, Sheng Wen, Junhui Zhang, Zhan Liao, Qian Zhang, Zhengjian Yan, Liangjun Yin, Jixing Ye, Youlin Deng, Hue H. Luu, Rex C. Haydon, Houjie Liang, Tong-Chuan He

Fig 2 is incorrect. It has recently come to the authors’ attention that there was an image assembling error for Fig 2B. Specifically, the image for “d8” group was inadvertently duplicated from that for “d7” group. The error does not affect the overall conclusion of our study, however, the authors apologize for any inconvenience caused by the error to the readers and the scientific community. The authors have provided a corrected version here.

**Fig 2:**
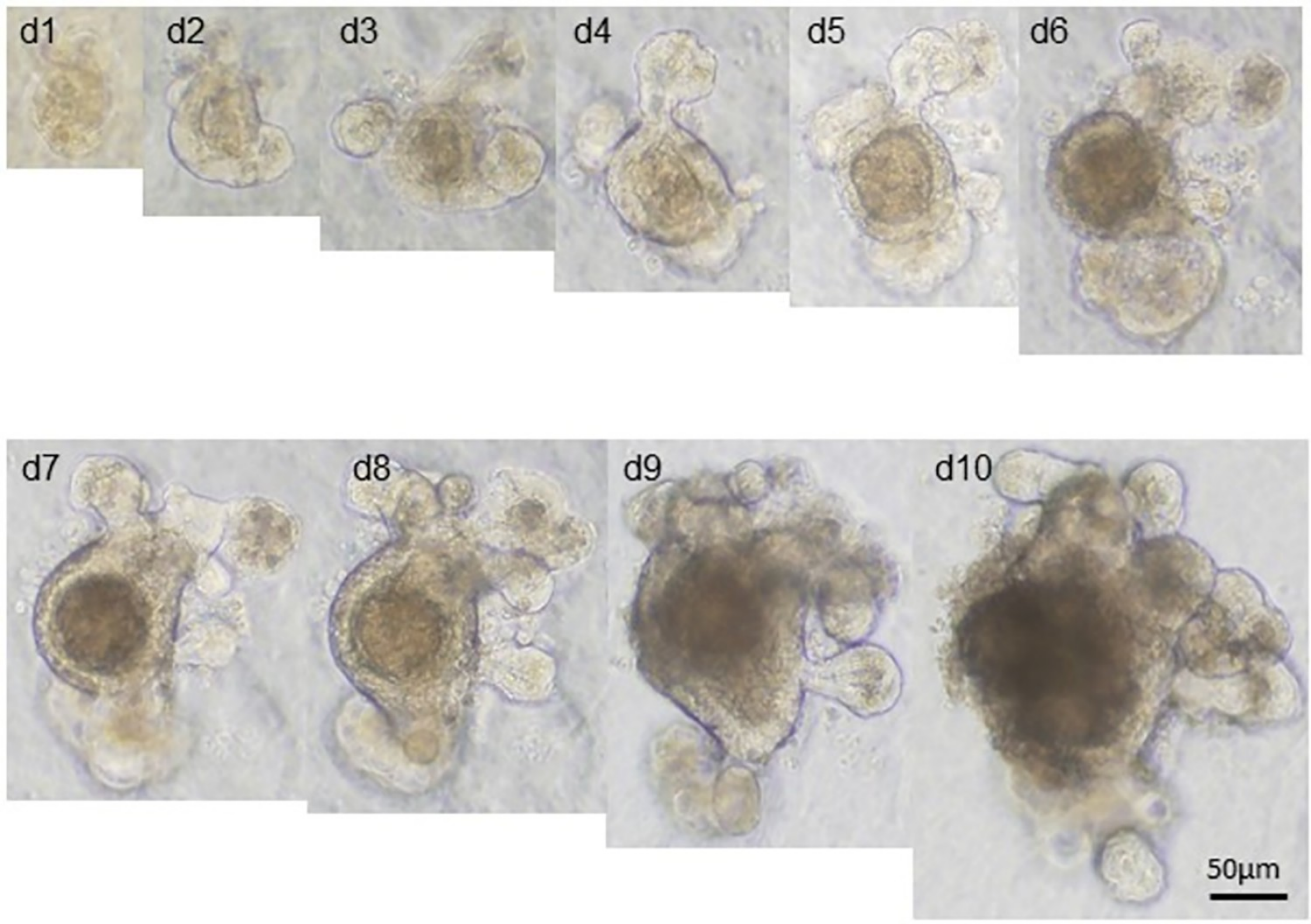
Three-dimensional morphology of the cultured “mini-gut” organoids. (A) Z-stack serial images of a multi-budding organoid. The bottom-to-top distance was 225 μm with 25 μm distance interval between images. (B) Monitoring the growth of a single organoid over a period of 10 days in 3-D Matrigel culture. Representative images are shown.
