# Supplementary figures and images for: Adenovirus-Mediated Efficient Gene Transfer into Cultured Three-Dimensional Organoids
Source: PLoS One. 2014 Apr 2;9(4):e93608. doi: 10.1371/journal.pone.0093608 (PMC3973564; doi:10.1371/journal.pone.0093608)

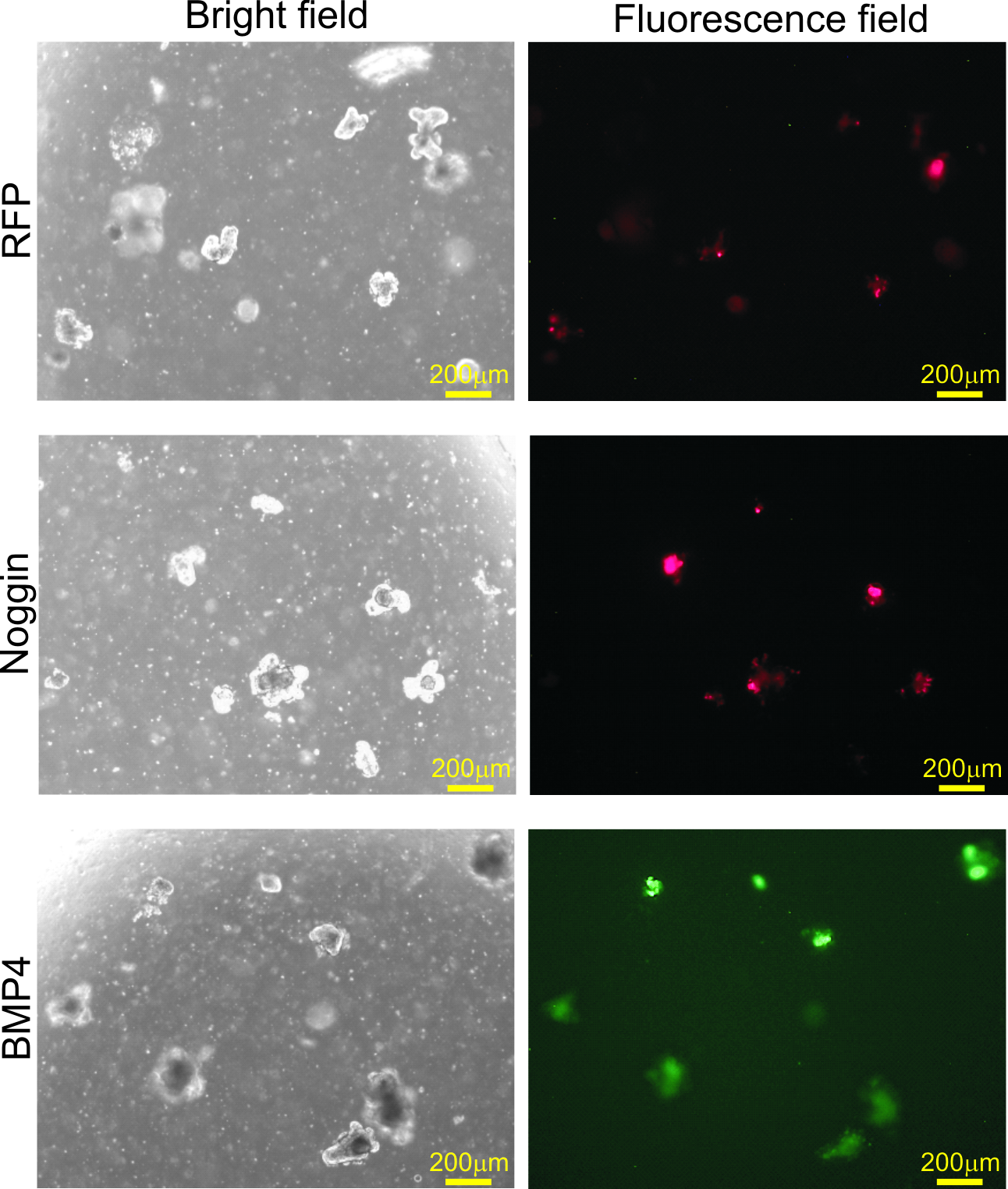

Supplement: Figure S1 — Efficient transduction of mini-gut organoids by adenoviral vector expressing noggin, BMP4, GFP, and/or RFP. 5×10^5^ pfu viral particles of Ad-Noggin (also expressing RFP), Ad-BMP4 (also expressing GFP), and Ad-RFP were added to the ice-chilled organoids Matrigel mix. The infected organoids were followed for fluorescence signal at 48 h after infection. Representative results are shown. (TIF) [file pone.0093608.s001.tif]
